# Can widely used cell type markers predict the suitability of immortalized or primary mammary epithelial cell models?

**DOI:** 10.1186/s40659-015-0063-2

**Published:** 2016-01-06

**Authors:** Edgar Corneille Ontsouka, Janique Sabina Bertschi, Xiao Huang, Michael Lüthi, Stefan Müller, Christiane Albrecht

**Affiliations:** Faculty of Medicine, Institute of Biochemistry and Molecular Medicine, University of Bern, Buehlstrasse 28, 3012 Bern, Switzerland; Swiss National Center of Competence in Research, NCCR TransCure, University of Bern, Bern, Switzerland; Department of Clinical Research, Faculty of Medicine, University of Bern, 3010 Bern, Switzerland

**Keywords:** Cell characterization, Flow cytometry, Gene expression, Lactation marker, Mammalian, Mammary epithelial cell

## Abstract

**Background:**

Mammary cell cultures are convenient tools for in vitro studies of mammary gland biology. However, the heterogeneity of mammary cell types, e.g., glandular milk secretory epithelial or myoepithelial cells, often complicates the interpretation of cell-based data. The present study was undertaken to determine the relevance of bovine primary mammary epithelial cells isolated from American Holstein (bMEC_US_) or Swiss Holstein–Friesian (bMEC_CH_) cows, and of primary bovine mammary alveolar epithelial cells stably transfected with simian virus-40 (SV-40) large T-antigen (MAC-T) for in vitro analyses. This was evaluated by testing their expression pattern of cytokeratin (CK) 7, 18, 19, vimentin, and α-smooth muscle actin (α-SMA).

**Results:**

The expression of the listed markers was assessed using real-time quantitative PCR, flow cytometry and immunofluorescence microscopy. Characteristic markers of the mesenchymal (vimentin), myoepithelial (α-SMA) and glandular secretory cells (CKs) showed differential expression among the studied cell cultures, partly depending on the analytical method used. The relative mRNA expression of vimentin, CK7 and CK19, respectively, was lower (*P* < 0.05) in immortalized than in primary mammary cell cultures. The stain index (based on flow cytometry) of CK7 and CK19 protein was lower (*P* < 0.05) in MAC-T than in bMECs, while the expression of α-SMA and CK18 showed an inverse pattern. Immunofluorescence microscopy analysis mostly confirmed the mRNA data, while partly disagreed with flow cytometry data (e.g., vimentin level in MAC-T). The differential expression of CK7 and CK19 allowed discriminating between immortal and primary mammary cultures.

**Conclusions:**

The expression of the selected widely used cell type markers in primary and immortalized MEC cells did not allow a clear preference between these two cell models for in vitro analyses studying aspects of milk composition. All tested cell models exhibited to a variable degree epithelial and mesenchymal features. Thus, based on their characterization with widely used cell markers, none of these cultures represent an unequivocal alveolar mammary epithelial cell model. For choosing the appropriate in vitro model additional properties such as the expression profile of specific proteins of interest (e.g., transporter proteins) should equally be taken into account.

## Background

The mature mammary gland (MG) is an exocrine organ that contains diverse mammary cell types bearing different morphology and organization. The apical glandular secretory epithelial cells form the inner layer of the branching ductal-lobular tree, which are lined by an outer layer of basal myoepithelial (or contractile) cells. The latter contract under oxytocin action and allow milk release. Stem cells of the basal layer exhibit high self-renewal and multi-lineage differentiation capacities [1–4]. It is documented for instance that a single stem cell can generate the entire functional ducto-lobular tree [5, 6]. Currently, primary mammary epithelial cells (MEC) isolated from MG tissues [4, 7, 8] or prepared from milk [9], and bovine mammary cell lines obtained by transfection of mammary alveolar cells with simian virus-40 (SV-40) large T-antigen such as (MAC-T) [10], HH2a [11] or BME-UV [12] are cell models that can be used for in vitro investigation of MG biology. MAC-T cells are described as retaining the ability to synthesize milk components such as caseins [10].

Cell-based experiments using mammary cell retaining milk secreting properties may serve to better understand the determinants of milk composition, and may contribute to the development of cellular and molecular strategies allowing the manipulation of milk composition for health and nutritional purposes [13]. However, a potentially important flaw in using MEC cultures is likely associated with heterogeneity and plasticity of MEC [14–16], which could complicate data interpretation. While the heterogeneity of primary mammary cultures is known due to proliferation of other mammary cell types (e.g., myoepithelial cells), in a recent study also the homogeneity of MAC-T has been questioned [17]. In this context, it appears important to evaluate the relevance of MAC-T and primary cells for in vitro studies on MG physiology by monitoring their expression level of marker antigens characteristic for different mammary cells e.g., luminal milk producing cells or mesenchymal cells.

The morphology and organization of mammalian cells including MEC are maintained by actin microfilaments and microtubules, which together with intermediate filament proteins constitute the components of the cell cytoskeleton. Accordingly, vimentin and cytokeratins (CKs) are often used as cell type markers [16, 18]. Based on that, the aim of this study was to characterize the suitability of selected primary and MAC-T cell cultures for in vitro studies on MG biology by measuring the expression of vimentin, α-SMA, CK7, CK18 and CK19 at the transcriptional and protein levels using different analytical methods. We hypothesized that vimentin and α-SMA—markers for mesenchymal and myoepithelial cells [16, 19]—are not expressed in cell cultures expressing CK proteins (e.g., CK18 and CK19), which are markers of alveolar milk secretory epithelial cells [20].

## Results

### Cell type marker mRNA expression

**Fig. 1:**
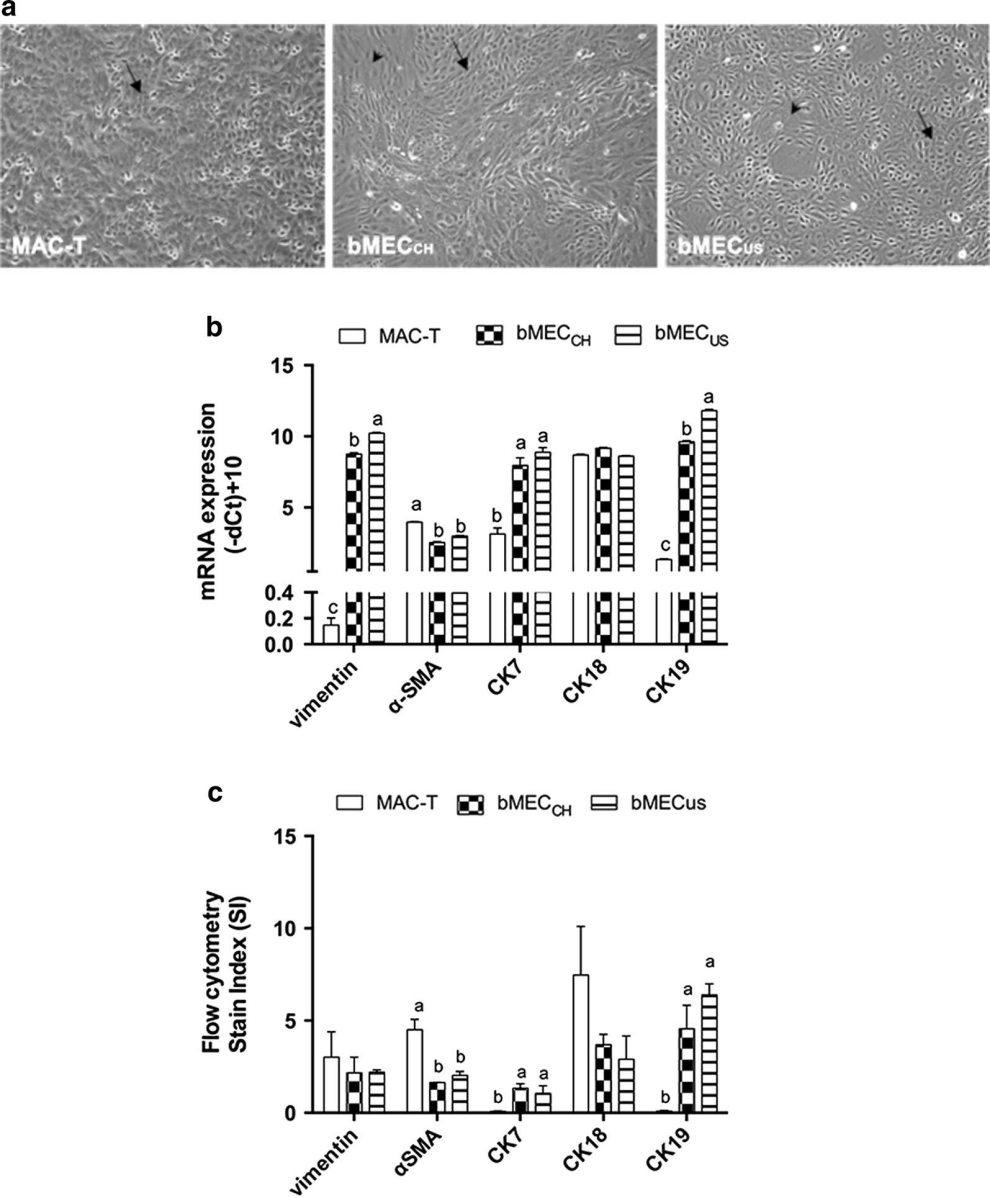
**a**. Morphology of bovine mammary cell cultures. The confluent monolayers of the bovine mammary epithelial cells were cultured as described in the text. *Black arrows* show characteristic cobblestone epithelial cells predominantly present in the monolayer. *Black arrowheads* depict mesenchymal-like cells. MAC-T: immortalized mammary epithelial cell line, 10× magnification; bMEC_CH_: bovine primary mammary epithelial cells isolated from a Swiss Holstein–Friesian cow at late lactation; 10× magnification; bMEC_US_: bovine primary mammary epithelial cells isolated from an American Holstein at mid-lactation, 10× magnification. **b**. The mRNA abundance of the selected markers of mesenchymal-like and epithelial cells in human and bovine mammary cell cultures. The gene expression of vimentin, α-smooth muscle actin (α-SMA), cytokeratin (CK) 7, CK18 and CK19 in MAC-T, bMEC_CH_, and bMEC_US_ was normalized to the mean of beta actin and ubiquitin. Details on the origin of the mammary cell cultures are described in **a**. Data are shown as mean ± SD of the (−delta Ct) + 10. The values are proportional to the gene expression level. *Bars* indicate the standard deviation of three independent experiments measured at least in duplicates. Different *letters* (a–c) indicate significant differences (*P* < 0.05). **c**. Protein expression of cell markers using flow cytometry. The protein expression of vimentin, α-SMA, CK7 and CK18 was expressed by using the Stain Index (SI) as described elsewhere [41]. SI = [median fluorescence intensity of positive (MFI) –MFI of negative]/(2 × SD of MFI negative). The MFI was derived from evaluation of flow cytometry data with FLOWJO Data Analysis Software. Data are expressed as mean ± SD of 2–3 independent experiments. Different *letters* (a, b) indicate significant differences (*P* < 0.05)

Quantitative RT-PCR analysis of confluent MAC-T, bMEC_CH_ and bMEC_US_ (Fig. 1a) revealed that the expression of reference genes did not vary among cell models. The corresponding mean Ct values were 13.1 ± 0.16, 12.6 ± 0.25 and 12.7 ± 0.15, respectively. As shown in Fig. 1b a detectable but, compared to CK18, a markedly lower normalized (relative) mRNA abundance of vimentin, α-SMA, CK7 and CK19 was detected in MAC-T. In parallel, the relative mRNA abundance of vimentin was greater (*P* < 0.05) in bMECs than in MAC-T (Fig. 1b). There were approx. ten Cts difference concerning vimentin expression between MAC-T and bMECs. The mean Ct values of vimentin amplification were 23.0 ± 0.19, 13.8 ± 0.21, and 12.5 ± 0.24 in MAC-T, bMEC_CH_ and bMEC_US_, respectively. Regarding α-SMA, the relative abundance of the corresponding mRNA transcripts was (unexpectedly) greater (*P* < 0.05) in MAC-T than in bMECs. On the other hand, the assessment of CK7 and CK19 indicated a markedly lower relative mRNA expression level in MAC-T (*P* < 0.05) compared to bMECs (Fig. 1b). The mean Ct values of CK7 and CK19 in MAC-T, bMEC_CH_ and bMEC_US_ were 20.0 ± 1.01, 21.8 ± 0.11, 14.6 ± 1.35 (CK7) and 12.9 ± 0.36, 13.9 ± 0.81, 10.9 ± 0.34 (CK19), respectively.

### Cell type marker protein expression

#### Flow cytometry analysis

Concerning the expression of characteristic markers of mesenchymal cells, the SI value of vimentin did not differ among all three mammary cell cultures, while that of α-SMA was significantly higher in MAC-T than in bMECs (Fig. 1c).

Regarding the CK proteins, the SI values of CK7 and CK19 were significantly higher in bMECs than in MAC-T. However, CK18 expression in MAC-T did not significantly differ between MAC-T and bMECs (Fig. 1c).

**Fig. 2:**
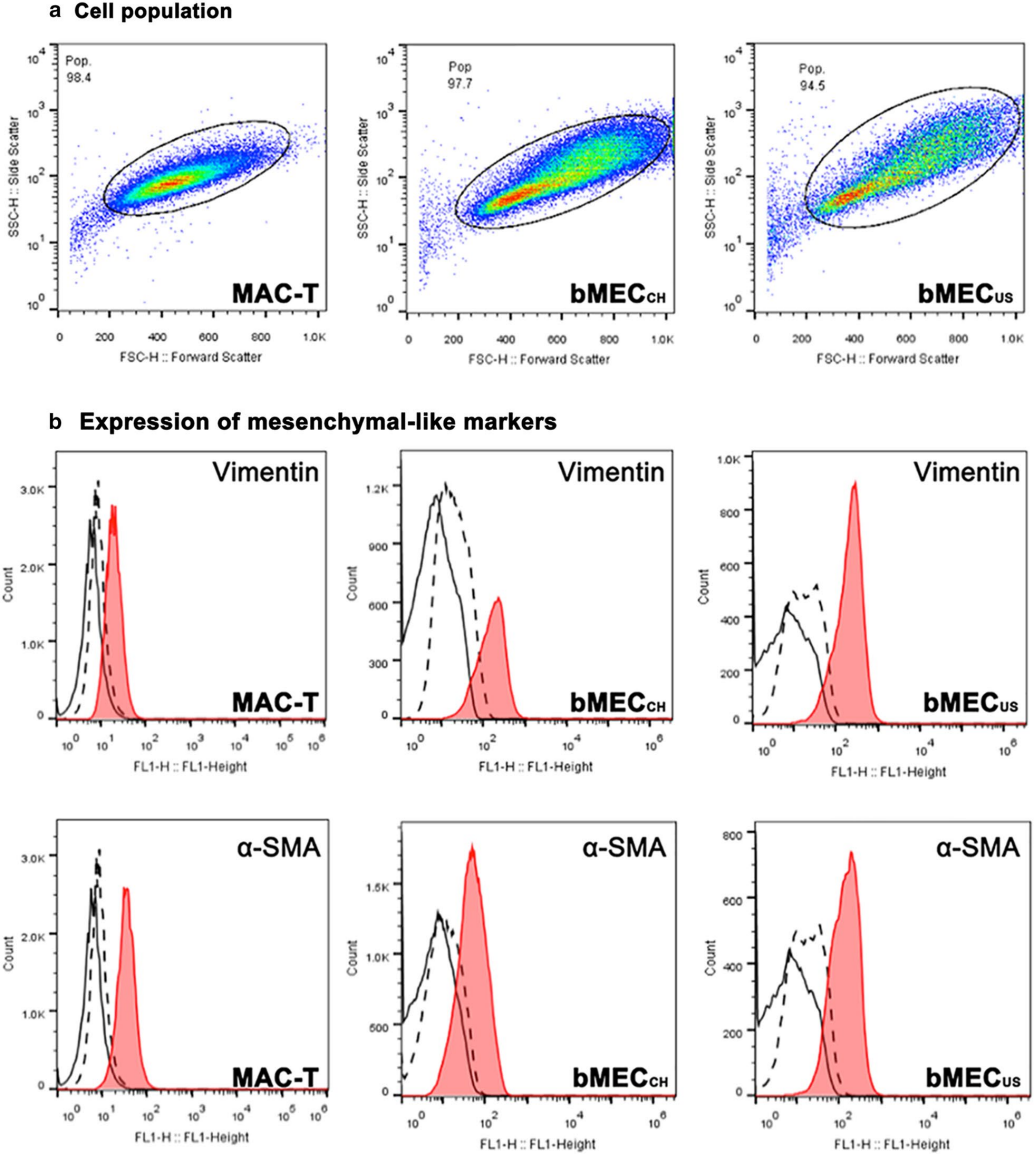
Flow cytometry analysis of selected markers of mesenchymal-like cells in primary and immortalized mammary cultures. **a**. Cell distribution pattern in MAC-T, bMEC_US_ and bMEC_CH_ cultures based on the forward scatters. **b**. The cell staining for vimentin and α-smooth muscle actin (α-SMA) in MAC-T, bMEC_US_ and bMEC_CH_ cultures, respectively, is shown in *red*. Two sets of control stainings were included: (i) without primary and secondary Ab (*small dashed lines*) and (ii) with IgG1 isotype and secondary Ab (*large dashed lines*). The staining was acquired by counting at least 20,000 events

**Fig. 3:**
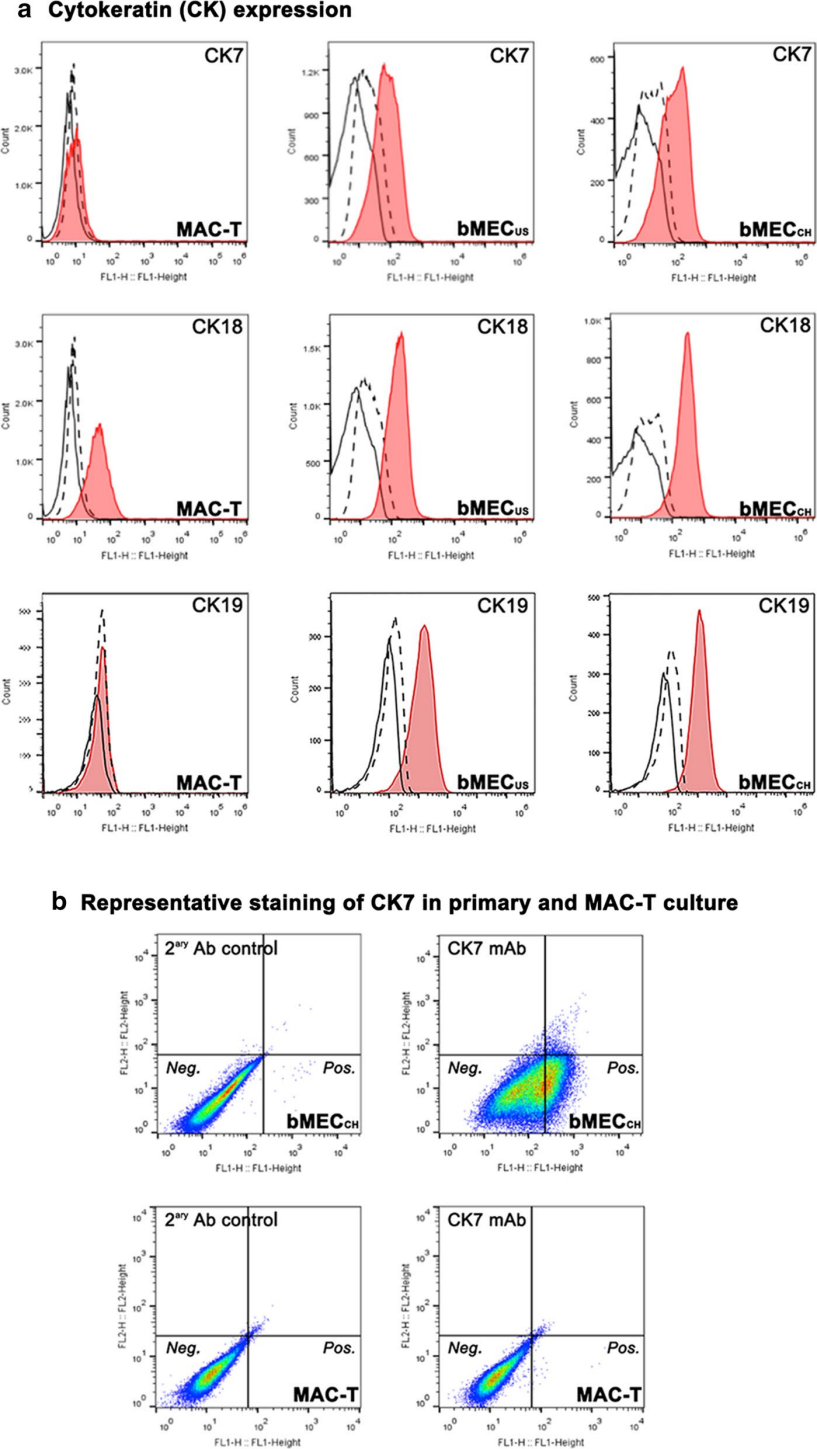
Flow cytometry of selected markers of epithelial cells in bovine primary and immortalized cell cultures. The cell populations are identical to the ones shown in Fig. 2a. **a**. The cell staining for cytokeratin (CK) 7, CK18 and CK19 in MAC-T, bMEC_US_ and bMEC_CH_ cultures, respectively, is shown in red. **b**. Distribution of CK7 positive cells in bMEC_CH_ and MAC-T cultures. The *X-axis* (FL1-Height channel) detects FITC-tagged antibody, while *Y-axis* (FL2-Height) detects Cy3-tagged antibody. All other details are as described in Fig. 2

As expected, the light scattering properties assessed by flow cytometry indicated more homogenous cell size and shape in MAC-T culture as compared to a wider distribution pattern seen from primary cell cultures (Fig. 2a). As compared to negative controls, there was a clear shift indicating positive staining of MAC-T cells for vimentin, α-SMA, and CK18 (Figs. 2b, 3a). In contrast, MAC-T cells were CK7- and CK19-negative (Fig. 3). Primary bMECs exhibited, unlike MAC-T, positivity for all tested markers (Fig. 2b and Fig. 3). Interestingly, the CK7 staining revealed the existence of two subpopulations (Fig. 3b), which were not detectable with CK18, a similar marker for epithelial cells (data not shown).

A summary of the flow cytometry results including the percentages of positive cells for vimentin, α-SMA, CK7, CK18 and CK19 is shown in Table 1.

**Table 1:** Summary of the flow cytometry analyses of selected cell type markers

Cell model	Passage no	Percentage of positive cells				
		Vimentin	α-SMA	CK7	CK18	CK19
MAC-T	15–22	79–93 %	98–100 %	0.4–0.9 %	92–99 %	0.1–0.2 %
bMEC_US_	12–15	51–87 %	56–64 %	35–50 %	70–88 %	92–96 %
bMEC_CH_	9–12	59–81 %	69–83 %	61–62 %	90–95 %	75–85 %

Values show ranges of the percentage of positively stained cells. Data are derived from two to three independent measurements for each cell model. The fluorescence intensity corresponds to the intensity of FITC conjugated polyclonal goat anti-mouse IgG Ab (BioLegend) positively reacting with mouse anti- α-smooth muscle actin (α-SMA) mAb (Novus Biologicals), anti-vimentin mAb (Sigma), anti-cytokeratin (CK) 7 (Dako), anti-CK18 mAb (Sigma), and anti-CK19 (Abcam), respectively. The staining was acquired by counting a minimum of 15,000 events. An IgG_1_ isotype control staining has been performed to ascertain the reliability of the positive staining. The background fluorescence corresponds to the intensity of FITC conjugated polyclonal goat anti-mouse IgG mAb staining in the presence of the isotype control IgG1 mAb (DakoCytomation,)

*MAC-T* immortalized bovine mammary epithelial cell line, *bMEC*_*US*_ bovine primary mammary epithelial cells isolated from an American Holstein cow at mid-lactation, *bMEC*_*CH*_ bovine primary mammary epithelial cells isolated from a Swiss Holstein–Friesian cow at late lactation, *Ab* antibody

#### Confocal microscopy

**Fig. 4:**
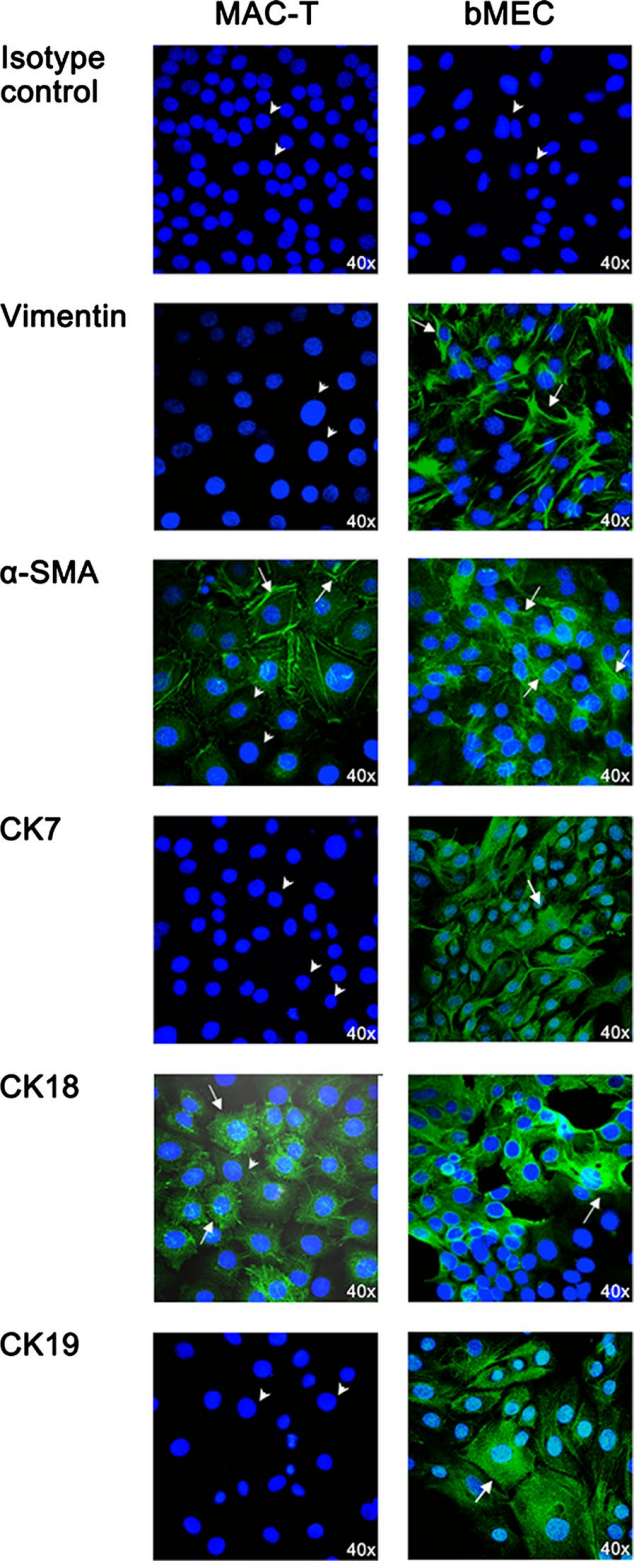
Fluorescence microscopy of selected cell markers in bovine mammary cell cultures. The figure shows representative fluorescence microscopy staining of bovine immortalized cell culture (*left* panel) and primary cell culture (*right* panel) for vimentin, α-smooth muscle actin (α-SMA), cytokeratin (CK) 7, CK18 and CK19. The negative isotype control IgG_1_ in each of cell culture is also shown. *White arrows* show positively stained cells whereas the *white arrowheads* indicate unstained cells. The fluorescence images were taken with the immunofluorescence microscope Nikon EZ-C1

As there was no statistical difference regarding the SI of tested traits between bMEC_CH_ and bMEC_us_, immunofluorescence microscopy was performed only in one primary cell type (bMEC_CH_). Regarding vimentin, the immunofluorescence staining indicated the presence of vimentin-positive cells in bMEC_CH_ but not in in MAC-T cultures (Fig. 4). Unlike vimentin, α-SMA-positive cells were identified both in bMEC_CH_ and MAC-T (Fig. 4). The α-SMA-positive staining in primary culture suggested the presence of microfilament-like structures diffusely located in the cytoplasm (Fig. 4). In contrast, microfilament structures in MAC-T were close to the cell borders (membranes) (Fig. 4). Regarding CK18, there was a more perinuclear localization of CK18 in MAC-T in contrast to the stronger diffuse CK18 staining in bMEC_CH_ (Fig. 4, right panel). CK7-positive and CK19-positive cells were not detectable in MAC-T cells while there was a diffuse staining with some membrane accentuation in bMEC_CH_ (Fig. 4).

## Discussion

The present study aimed at a thorough cell marker-based characterization of bovine primary and immortalized MEC cultures using different methodological and analytical approaches (real-time PCR, flow cytometry and immunofluorescence microscopy). Current literature indicates that vimentin and α-SMA are useful markers for mesenchymal cells, while, for instance, CK7, CK18 and CK19 are characteristic markers for mammary alveolar secretory epithelial cells [16, 20–23]. On the other hand, the established MAC-T cells have been described as retaining the ability to synthesize milk-related components such as α_s_ caseins [10]. Indeed, we observed an increase of the mRNA transcripts of α_s1_ casein and α-lactalbumin in MAC-T under certain experimental conditions, supporting the presence of fully differentiated alveolar secretory cells in MAC-T culture (Bertschi, Ontsouka and Albrecht, unpublished data). On this basis, we hypothesized that the expression of characteristic markers for mesenchymal and myoepithelial cells (vimentin and α-SMA, respectively) will be marginal or absent in MAC-T whereas CK7, CK18 and CK19 were expected to be highly expressed.

Concerning the expression of vimentin, this assumption seemed to be confirmed by immune detection with confocal microscopy. Indeed, unlike primary cells tested at passages 9–15, vimentin positive signals were not found in MAC-T cells analyzed at passages 15–22. The absence of vimentin staining in MAC-T cells shown by confocal microscopy was in accordance with earlier investigators who failed to detect vimentin staining in MAC-T cells by phase contrast imaging [17]. However, the Ct values obtained by qPCR amplification indicated the presence of vimentin, although in variable abundance, in all cell cultures studied. The normalized vimentin mRNA expression in MAC-T was low as compared to primary cells (approx. ten Cts difference). In contrast to the data at the transcriptional level, the obtained flow cytometry SI values showed similar expression levels of vimentin in primary and MAC-T cells. This discrepancy may be at least partly explained by variable mRNA stability and turnover rates between primary and immortalized cells. On the other hand, since antibodies used for flow cytometry and confocal microscopy analyses were identical, it is likely that also different sensitivities and detection limits of these two immunologically based techniques play a role. In this regard, confocal microscopy detects signal from cells fixed on slides, while flow cytometry is a single cell-based detection with higher degree of statistical precision [24]. These findings imply the need of using standardized methods for a reliable cell characterization, which reduce the risks of possible misleading outcomes and interpretations of results (as stated above for vimentin).

α-SMA it is a typical marker for myoepithelial and mesenchymal cells [16, 19] which are also present in the MG. Considering flow cytometry light scattering properties of MAC-T and primary cells, respectively, and knowing that MAC-T cells retain milk secretory characteristics [10, 25, 26], we expected a lower mRNA and protein expressions of α-SMA in MAC-T than primary cells. Surprisingly, the expression of α-SMA in primary cells was lower than in MAC-T cells. An even lower α-SMA expression was observed in human primary MEC tested at passage 4 (unpublished data).

It is worth to note that expression of α-SMA has been described in cells other than pure muscle cells such as myofibroblasts which are also present in MG tissues [27, 28]. The (de) differentiation of mammary alveolar epithelial cells to myoepithelial cells with a gradual gain of myoepithelial markers (e.g., α-SMA expression) has been demonstrated in in vitro studies [20, 29, 30]. Based on that, the relatively higher α-SMA expression in MAC-T compared to primary cells can reflect differences in the proportion of epithelial cells undergoing dedifferentiation, thereby expressing α-SMA. In the current study, the microfilaments were localized beneath the plasma membrane in MAC-T while they showed a cytoplasmic distribution in primary cells. The distribution pattern observed in MAC-T agreed with findings reporting the presence of actin-like filaments beneath the plasma membrane in close association with secretory vacuoles in lactating guinea pig mammary epithelial cells [31].

The process of mammary cell (de) differentiation—named epithelial-to-mesenchymal transition (EMT)—occurring within the MG [14, 15, 21] might be more pronounced in MAC-T culture due to cell immortalization procedures. Indeed, a study investigating hepatocytes, another type of epithelial cells which was also transfected with SV40 large T antigen, has reported an increase of epithelial cell dedifferentiation into the mesenchymal phenotype [32]. Regarding cell transition, vimentin expression in epithelial cells is believed to influence and illustrate the transition from epithelial to mesenchymal phenotypes [16]. Conversely, overexpression of a dominant-negative mutant or the silencing of vimentin (neither of which alter microtubule or microfilament assembly) has been shown to cause mesenchymal cells to adopt epithelial shapes [16, 33]. In the current study, the percentage of vimentin positive cells was between 79 and 93 % in MAC-T, while it oscillated between 51 and 87 % in primary cells. This suggests that epithelial cell transition to the mesenchymal phenotype probably occurs in all these cell cultures. In addition, as stated above, the expression of mRNA transcripts encoding for α_s1_ casein, a hallmark of fully differentiated mammary alveolar cells, was induced in MAC-T medium containing insulin, epidermal growth factor and progesterone (Bertschi, Ontsouka and Albrecht, unpublished data). Conversely, the mRNA abundance of α_s1_ casein was upregulated by 7.8–13.5-fold in primary cells treated with lactogenic hormones for 9–14 days (Bertschi, Ontsouka and Albrecht, unpublished data). These observations suggest the suitability of both primary and immortalized cell models for in vitro studies related to specific aspects of MG biology.

Concerning CK markers, CK18 and CK19 are considered as gold markers for luminal (milk producing) cells [20, 23, 30]. Evidences from in situ staining demonstrated that CK7 has predominantly luminal expression in human mammary tissues [34]. In this study, we consistently found that, in contrast to primary cells, CK7 and CK19 levels were very low or undetectable in MAC-T. The lack of CK7 expression was also found in MCF-7 cells, a human breast adenocarcinoma derived cell line (unpublished data). These findings suggest that, compared to immortalized cells, only primary mammary cells would contain amounts of glandular milk producing cells. However, unlike CK7 and CK19, we found that the expression level of CK18, also an important marker of luminal cells, was comparable in MAC-T and primary cells. The detection of CK18 in primary and immortalized MAC-T cells argues for the presence of luminal milk producing epithelial cells. Indeed, CK18 is most prominent in the lumen lining cells (for review see [23]) and its usefulness as a characteristic marker for mammary luminal cells has been also confirmed in bovine mammary tissues [35]. In summary, the apparent contradiction between and within the expression patterns of CKs and mesenchymal markers tested in this study underlines the complexity of mammary derived cells and the risk of determining cell suitability based solely on the analysis of cell type markers’ expression. The findings reported in the current study suggest, however, that CK7 and CK19 are reliable cell markers to efficiently discriminate between primary and immortalized cell cultures.

## Conclusions

The expression of the selected widely used cell type markers in primary and immortalized MEC cells did not allow a clear preference between these two cell models for in vitro analyses studying aspects of milk composition. All tested cell models exhibited to a variable degree epithelial and mesenchymal features. Thus, based on currently widely used cell markers none of these cultures represent an unequivocal alveolar mammary epithelial cell model. For choosing the appropriate in vitro model additional properties such as the expression profile of specific proteins of interest (e.g., nutrient transporter proteins, signaling molecules) should equally be taken into account.

## Methods

### Bovine MEC

Bovine primary mammary epithelial cells isolated separately at mid and late lactation from two American Holstein **(**bMEC_US_) cows and one Swiss Holstein–Friesian (bMEC_CH_) cow have been previously described [4, 36, 37]. The immortalized bovine mammary cell line (MAC-T) has been established decades ago by transfecting primary bovine mammary epithelial cells with SV-40 T-antigen. These cells are believed to maintain milk secretory characteristics [10, 26]. MAC-T was provided by Dr. Laura Hernandez, University of Wisconsin at passage 3. bMEC_US_ and bMEC_CH_ were obtained from Dr. Craig Baumrucker, Penn State University and Dr. Olga Wellnitz, University of Bern at passages 4 and 1, respectively. Frozen cells (bMEC_CH,_ passage 2; bMEC_us_, passage 7; MAC-T, passage 8) were thawed and cultured in culture media at 37 °C with 5 % CO_2_ in T75 polystyrene culture flasks as described below. MAC-T cells were cultured for 3–5 days until confluence, while bMEC were cultured for 5–7 days prior to reach confluency (Fig. 1a). All cell types (bMEC_CH_, bMEC_US_ and MAC-T) were cultured in Dulbecco’s modified Eagle’s medium (DMEM)-F12 medium (Gibco) supplemented with 10 % fetal bovine serum (FBS), 1 % (v/v) antibiotics/antimycotics’ solution containing penicillin, 100 units/ml; streptomycin, 100 µg/ml; and amphotericin B, 0.25 µg/ml (Sigma). In addition, cells were supplemented with 1× ITS mixture (Sigma, Buchs, Switzerland) composed of insulin, transferrin and sodium selenite at the final concentrations of 10 ng/mL, 5.5 ng/mL and 6.7 pg/mL, respectively. For routine passaging, MEC were treated with 0.05 % trypsin–EDTA (3–5 ml/75 cm^2^flask) until cells detached.

### Quantitative RT-PCR

For RNA extraction, as well as other hereafter described analyses, confluent and MAC-T, bMEC_CH_ and bMEC_US_ were used at passages between 15–22, 9–12 and 12–15, respectively (Fig. 1a). The procedures, materials, and reagents used for the reverse transcription and Sybr green qPCR have been previously described [38]. In brief, the SYBR Green dye-based real-time quantitative PCR measurements have been performed by using the GoTaq^®^qPCR Master Mix (Promega). The amplification reactions were performed using 75 ng reversed transcribed RNA in duplicates on 384-well plates (Applied Biosystems) on the ViiA7 (Applied Biosystems) using default cycling conditions for 40 cycles: activation of enzyme at 95 °C/10 min, denaturation at 95 °C/15 s, annealing at 60 °C/1 min, followed by the melting step. The melting curves were analyzed for the absence of additional PCR products or primer dimers. The primer pairs, partly obtained from previous studies [39], used for the PCR amplification of α-SMA, vimentin, CK7, CK18, CK19, beta actin, and ubiquitin are summarized in Table 2. The relative mRNA expression (delta Ct; dCt) of cell type markers was obtained by relating their respective Ct values to the mean Ct values of beta actin and ubiquitin as follows: dCt = Ct target—Ct mean housekeeping genes. A lower dCt value corresponds to higher mRNA expression. As described elsewhere [40], the results were then expressed as (−dCt) + 10. This allows an easy interpretation of results because the higher value corresponds to higher mRNA expression.

**Table 2:** Primer pairs used for gene amplification in bovine mammary epithelial cells

Gene	Accession number	Primer pairs (5′- end to 3′- end)	Product length (bp)
α-SMA	NM_001034502	For: GGTGATGAAGCACAAAGCAA Rev: TGAGAAGGGTTGGATGCTCT	154
Vimentin	NM_173969.3	For: CGCTCAAAGGGACTAACGAG Rev: TGACATTCAGCAGGTCTTGG	174
CK7	NM_001046411.1	For: TTACCAGACCAAGTTTGA Rev: ATCTCATTCCGGGTATTC	78
CK18	NM_001192095.1	For: ATTGATAATGCCCGTCTTGC Rev: AGCCTCGATCTCAGTCTCCA	156
CK19	NM_001015600.3	For: GATGACTTCCGCACCAAGTT Rev: AGCAGAATCCACCTCCACAC	234
Beta actin	XM_006715764.1	For: AACTCCATCATGAAGTGTGACG Rev: GATCCACATCTGCTGGAAGG	234
Ubiquitin	see [39]	For: TTCACAGGTCAAAATGCAGA Rev: ATCTGCATACCACCCCTCAG	237^a^

^a^Primers are obtained from the above mentioned studies

*Bp* base pairs, *CK* cytokeratin

### Flow cytometry

Trypsinized from one T75 flask were washed with precooled dPBS (Life Technologies, Zug, Switzerland) and centrifuged at ~80*g* for 5 min. For fixation and permeabilization, cells were resuspended in 1 ml of 100 % (v/v) methanol (stored at −20 °C) and kept on ice for 10 min. Thereafter, cells were washed and resuspended in dPBS supplemented with 10 % (v/v) FBS. For staining, cells were incubated for 1 h on ice with either mouse anti-vimentin mAb (1:500; Sigma, Saint Louis, Missouri), anti-CK7 mAb (1:150; Dako, Glostrup, Denmark), anti-CK18 mAb (1:500; Sigma, Saint Louis, Missouri), anti-CK19 mAb (1:200; Abcam) or anti-actin α-smooth muscle mAb (1:200; Novus Biologicals, Cambridge, United Kingdom). In addition, the staining of an isotope control IgG_1_ at dilution 1:200 (DakoCytomation, Glostrup, Denmark) was performed to ascertain the specificity of all measured signals. After washing the cells twice with dPBS supplemented with 10 % (v/v) FBS, they were incubated on ice for 1 h in the dark with FITC conjugated polyclonal goat anti-mouse IgG antibody diluted 1:500 (BioLegend, San Diego, California). After two additional washing steps with dPBS supplemented with 10 % (v/v) FBS, stained cells were resuspended in dPBS and kept in the dark until analysis. For each staining, at least 20,000 events were acquired on the FACScan flow cytometer BD Instruments (San Jose, CA). Data were analyzed with FLOWJO Data Analysis Software (Tree Star Inc, Ashland, OR). The protein expression of cell type markers was expressed as the stain index (SI) using the following equation: SI = (median fluorescence intensity (MFI) of positive—MFI of negative)/(2 × standard deviation of MFI of negative) [41]. The stain index allows normalizing the protein expression against the differential background signal from cells in the respective cultures.

### Immunofluorescence microscopy

Cells grown on coverslips in a 12-well plate were fixed and permeabilized with 1 ml of 100 % (v/v) methanol as described above. Thereafter, they were washed three times (5 min each) with chilled dPBS and three more times with cold dPBS supplemented with 0.1 M glycine. For staining, cells on the coverslips were first blocked with dPBS containing 2 % (w/v) BSA and 4 % (v/v) goat serum for 1 h at room temperature. Then, cells were incubated for 2 h at room temperature with mouse anti-vimentin mAb, anti-α-SMA mAb, anti-CK18 mAb, and anti-CK19 prepared in dPBS containing 1 % (w/v) BSA and 2 % (v/v) goat serum at the respective dilutions described above for flow cytometry. After washing the cells three times with dPBS, they were co-incubated at room temperature for 1 h in the dark with DAPI of a 1:25000 dilution and FITC conjugated polyclonal goat anti-mouse IgG antibody at the same dilution as described for flow cytometry. The images of cell staining were taken with the immunofluorescence microscope Nikon EZ-C1.

### Statistical analysis

The statistical evaluation of data was performed using GraphPad Prism software (San Diego, CA). All data are shown as mean ± SD. The differences in the mRNA abundance and SI values of selected cell type markers among mammary cell cultures were determined using two-way ANOVA with Tukey multiple comparisons *t* test. The level of statistical significance was set at *P* value < 0.05.

## Abbreviations

CK: cytokeratin
MEC: mammary epithelial cells
MG: mammary gland
SI: stain index
SMA: smooth muscle actin
